# Improved Algorithm for Analysis of DNA Sequences Using Multiresolution Transformation

**DOI:** 10.1155/2015/786497

**Published:** 2015-04-27

**Authors:** T. M. Inbamalar, R. Sivakumar

**Affiliations:** Department of Electronics and Communication Engineering, RMK Engineering College, Chennai 601206, India

## Abstract

Bioinformatics and genomic signal processing use computational techniques to solve various biological problems. They aim to study the information allied with genetic materials such as the deoxyribonucleic acid (DNA), the ribonucleic acid (RNA), and the proteins. Fast and precise identification of the protein coding regions in DNA sequence is one of the most important tasks in analysis. Existing digital signal processing (DSP) methods provide less accurate and computationally complex solution with greater background noise. Hence, improvements in accuracy, computational complexity, and reduction in background noise are essential in identification of the protein coding regions in the DNA sequences. In this paper, a new DSP based method is introduced to detect the protein coding regions in DNA sequences. Here, the DNA sequences are converted into numeric sequences using electron ion interaction potential (EIIP) representation. Then discrete wavelet transformation is taken. Absolute value of the energy is found followed by proper threshold. The test is conducted using the data bases available in the National Centre for Biotechnology Information (NCBI) site. The comparative analysis is done and it ensures the efficiency of the proposed system.

## 1. Introduction

The biological macromolecule, deoxyribonucleic acid (DNA), was discovered at the outset by Frederich Miescher in 1869 and the double helix structure of DNA was recommended in 1953 [1]. Owing to the sequencing of Bacterium [2], the genomes of a number of organisms have been fully sequenced. The sequence data of the DNA of organisms is accessible in databanks such as GenBank at the National Centre for Biotechnology Information (NCBI), DNA Database of Japan (DDBJ), and the European Bioinformatics Institute (EBI) [3–6]. Bioinformatics makes good judgement of the enormous amount of biological information produced by the human genome project. It uses computational techniques to comprehend the information allied with genetic materials such as deoxyribonucleic acid (DNA), ribonucleic acid (RNA), and proteins. This requires competent techniques to investigate and also to infer the outcome in a biologically significant manner [7].

Digital signal processing (DSP) is the mathematical operation on an information signal to alter or improve it using digital signal processors. It is characterized by the illustration of signals in discrete domain and the processing of these signals [8]. Since biological sequences are alphabetical in nature, they can be converted into numerical sequences and then DSP techniques can be applied for their investigation. Nowadays, computer algorithms based on digital signal processing are admired for understanding the characteristics of DNA, RNA, and protein sequences. Powerful signal processing techniques, for instance, transform methods and digital filters, are nowadays fruitfully applied to predict biologically noteworthy information on genomic sequences.

The genes in eukaryotic DNA have an alternating arrangement of protein coding regions (exons) and the noncoding regions (introns) of a gene. The identification of protein coding regions has helped genetic engineers to isolate proteins. This can also help in scheming personalized drugs for various diseases. Hence, the prediction of protein coding regions in DNA sequences is a key step in understanding genetic processes [9]. On the other hand, in eukaryotic DNA, the presence of protein coding regions and noncoding regions alternatively with uneven lengths is a hurdle in providing an accurate solution. It has been experimentally studied that the DNA segments related to protein coding regions have a tendency to display a strong spectral component at the frequency of 2*π*/3 called period-3 property [10–12]. Additionally, a long-range correlation exists in the genome sequence contributing background noise. This makes the job more complex [13]. It is important to note that the period-3 property is recognized by scientists and researchers as a good quality preliminary indicator of protein coding regions. In this paper, we discuss a method to locate protein coding regions by analyzing DNA sequences using digital signal processing.

The significant contributions of the paper are

- the paper discusses a method using genomic signal processing (GSP) to locate protein coding regions in DNA sequences;
- proposed method uses a joint time-frequency analysis, to analyze the spectrum obtained, and then it locates the protein coding regions;
- adaptive filtering is used to reduce the noise;
- compared with existing methods, our scheme is found to be efficient, reduced in noise, and better in detection of short exons.

The rest of this paper is organized as follows: Section 2 gives a brief description of the related works.  Section 3 explains in detail about the proposed method for location of the protein coding regions.  Section 4 describes the results and discussions.  Section 5 gives a brief summary of our overall work.

## 2. Review of Related Works

There have been many works in the literature associated with genomic signal processing. Numerical mapping is the first step in genomic analysis using digital signal processing. Locations of the protein coding regions are important for genome sequence analysis. Many computational approaches have been proposed and proved successfully in the past two decades for the detection of protein coding regions in DNA sequences. In this section, we discuss about few numeric representation schemes and some of the methods used for the location of protein coding regions in literature.

### 2.1. Numerical Mapping of DNA Sequences

The DNA sequence consists of four alphabets, namely, A, T, C, and G. The alphabet “A” is representing “Adenine,” “T” is representing “Thymine,” “C” is representing “Cytosine,” and “G” is representing “Guanine”. In order to apply suitable digital signal processing methods to DNA sequences, the character string of these sequences should be mapped to numerical sequences. This is done by assigning a numeral to every nucleotide that constitutes the DNA sequence. The objective of this mapping is to improve the hidden information with a view to promoting investigation. There are several mapping schemes found in the literature and a few among them are discussed here.

For numeric representation, a complex number mapping [10] has been suggested in which a particular complex number to each base is assumed given by A = 1 + *j*, T = 1 − *j*, C = −1 − *j*, and G = −1 + *j*. Voss mapping, or binary indicator sequences mapping [13], assigns a numeral “1” when a particular symbol is found in the sequence or else a “0.” A real number mapping [14] has been in use as if it is given by A = −1.5, T = 1.5, C = 0.5, and G = −0.5. A tetrahedron mapping [15] has been in use, in which the four alphabets are assigned to four corners of a regular tetrahedron. A three-dimensional curve representation called the *Z*-curve mapping [16] has been proposed for DNA sequences. Electron ion interaction potential (EIIP) values [17] for mapping DNA sequences have also been in use.

### 2.2. Location of Exons in a DNA Sequence

There are a number of methods found in literature for identifying exons in the DNA sequences. Some of the techniques based on genomic signal processing are summarized here.

An algorithm called “TESTCODE” [18] based on probability of coding has been proposed to predict protein coding regions in DNA sequence. The method is completely objective and does not require a computer. Based on the fact that Fourier spectrum exhibits peaks at *f* = 1/3, a computer programme “Gene Scan” [11] has been developed to locate coding open reading frames and protein coding regions in DNA sequences. This is done by examining the local signal to noise ratio of the peak within a sliding window. The method has been proved to be independent of training set, base compositional variations, and a priori knowledge of the sequences. A method based on digital filter has been introduced for identifying the protein coding regions [12]. The advantage of this method is elimination of the background 1/*f* spectrum. In addition, it is proved that multistage narrow band filters with pass band centered at 2*π*/3 are excellent in its action. A single digital filter followed by a quadratic window has been proposed [19] to suppress the noncoding regions in the DNA spectrum at 2*π*/3 and this method improves accuracy. Mutual information based tools [20] have been used for the identifying fragments of DNA principally well suited to determine short tandem repeats. An algorithm based on individual periodicity analysis of each nucleotide followed by their combination [21] to recognize the accurate and inaccurate repeat patterns in DNA sequences has been proposed. A method to identify protein coding regions in DNA sequences using statistically optimal null filters (SONF) [22] has been described. Voss representation is used for binary conversion and then processed by a separate SONF producing improved efficiency in predicting short exons.

An algorithm using short time Fourier transform (STFT) followed by windowing using the period-3 property [17] has been proposed and found to be efficient in detecting exons. An algorithm to identify protein coding regions using a modified Gabor wavelet transform [23] has been proposed. The method is independent of the window length and has enhanced identification accuracy. A method for protein coding region identification in the DNA by exploiting the period-3 property and autoregressive (AR) modeling [24] has been proposed. The advantage is less computation time. They have used S transform [25] for the same problem and obtained increased accuracy with the advantage of model independency. Mutual Information has been identified as the best parameter to discriminate coding and noncoding sequences of a DNA [26]. It has been found that for analyzing prokaryotic genome with very short length of noncoding sequences, probabilistic models such as Hidden Markov Model (HMM) or Bayesian Network are successful. For examining the position of exons in DNA strand, a new procedure based on autoregressive spectrum analysis [27] has been proposed. Wavelet packets are used to remove noise. This method used standard datasets and shows improved performance. An improved self-adaptive spectral rotation technique [28] has been suggested to get rid of the noise. This also visualises the triplet periodicity walks, persistency and antipersistency, and so forth.

From the survey, it is understood that the increase of computational techniques in finding the gene location in literature is pretty hopeful and successful, but the proficiency of the techniques needs to be improved. Furthermore, it is learnt that most of the approaches used period-3 property of the DNA sequence to detect the protein coding regions. Majority of the authors attempted to suppress the background noise in the DNA spectrum. In general eukaryotic DNA is taken as test sample for performance evaluation. The test is specifically done for the DNA sequence of gene* F56F11.4* of* Caenorhabditis elegans* chromosome III [Gene bank: AF099922].

## 3. The Proposed Wavelet Based Analysis

### 3.1. Motivation

Generally, the Fourier transform is determined by finding the time average of all the spectral components. But the spectral content of the signal varies with time. The Fourier transform produces details about the frequency contents, but it does not give details about the time of occurrence of that frequency content. Hence, the time aspects of the function disappear in the spectrum.

Gabor Transform (sliding window Fourier transform) provides time-frequency analysis. But as frequency increases, the temporal resolution is constant. Hence, there is a requirement of a signal analysis technique that could characterize the signal in both time and frequency domains locally and simultaneously. This type of spectrum analysis is known as time-frequency analysis. One such method is wavelet transform. Wavelet transform is a local transform, with variable temporal resolution capable of describing the local behaviour of signals on various time scales [29–32]. Hence, it is recommended to use wavelet filtering for locating the protein coding regions in DNA sequences. We consider the period-3 signal in the DNA sequence to be the signal of interest and the remaining are treated as noise. Hence, the wavelet filtering technique is used as a competent technique to take out the protein coding regions in the DNA segment.

### 3.2. Wavelet Transform

In wavelets, a family of functions is defined by dilation in scale and translation in time. Wavelets constitute a mathematical “zoom” making it possible simultaneously to describe the properties of signal on several timescales. They help to invent new techniques for signal analysis and other practical applications. Specifically, they excel in signal and image denoising or estimation of functions.

Denoising means recovering the useful signal from observed signal in which the useful signal is ruined by noise. Wavelet representations yield very simple algorithms for denoising applications. Due to their adaptability, they are easy to work with and are often more powerful compared with the conventional methods. The principle consists of wisely modifying the wavelet coefficients of observations and using thresholds. In order to avoid the existing redundancy in continuous wavelet transform and also to perform multiresolution analysis, discrete wavelet transform (DWT) is preferred.

DWT is a sampled version of the continuous case with discrete dilation and translation parameters. Filters or different cut-off frequencies are used to analyse the signal at different scales or resolution. Here scaling function corresponds to a low pass filter representing the approximate coefficients and the wavelet function corresponds to a high pass filter representing detail coefficients [31]. A fast algorithm of decomposition reconstruction for the discrete wavelet transform has been in use which is remarkably simple and its complexity is lower than that of FFT [32].

Wavelets with filters are associated with multiresolution orthogonal or biorthogonal analysis. Discrete transform and fast calculations using Mallat algorithm are then possible. Such wavelets with compact support available are Haar, Daubechies N, Symlet N, Coiflet N, which are orthogonal, and Biorthogonal N·N which is biorthogonal. Coiflets are compactly supported wavelets with highest number of vanishing moments for both phi and psi functions for a given support width. Symlets are compactly supported wavelets with low asymmetry and high number of vanishing moments for a given support width. Associated scaling filters for Symlets are near linear-phase filters. Biorthogonal wavelets are compactly supported spline wavelets for which symmetry and exact reconstruction are possible with FIR filters.

### 3.3. Identification of Protein Coding Regions Using Wavelet Based Filtering Approach

In this section, we describe the proposed system using wavelet transform methods. Here, we use discrete wavelet transform to solve the problem of protein coding region identification in DNA sequences.

The protein coding regions in DNA sequences are usually discontinuous and random in nature. Also, it is alternating between coding regions and noncoding regions. And, there are many nucleotides in the DNA strand. Hence, in order to locate the coding regions in a DNA sequence, many nucleotides which form the DNA strand have to be analyzed.

In DNA analysis, researchers have proved that the protein coding regions in a DNA sequence exhibit the period-3 property. But the noncoding regions do not possess this property. Hence, this property can be used as marker to determine the location of protein coding regions in a DNA sequence.

The DNA sequences are collected from the NCBI site. In order to apply digital signal processing techniques to DNA sequence, it has to be converted into numeric sequences. EIIP values as given in Table 1 have been used for the conversion. It is known that the EIIP sequence indicators have the following features:

- Average energy of delocalized electrons of the nucleotide is called an electron ion interaction potential. Hence, the EIIP of a nucleotide is a physical quantity.
- It is biologically more meaningful as it represents a physical property when compared to the indicator values which represent just the presence or absence of a nucleotide.
- It involves only a single sequence instead of four in the case of Voss representation thereby reducing the computational overhead by 75%.
- In literature, EIIP values have been successfully applied for analyzing DNA in numerous studies.
- EIIP values have been publicized to provide the most suitable mapping for spectral analysis of DNA sequences.

For these reasons, it has been proposed to use EIIP representation for numerical mapping of DNA sequence in our paper. The proposed DWT based computational process for location of exons using DWT approach is derived as given below.

Let the DNA string be represented by *D*[*n*] of length *k*, where it is made of A, T, C, and G. Consider

(1) $$\begin{array}{c} D\left[\;n\;\right]=\left\{\;\text{A},\text{T},\text{C},\text{G}\;\right\}. \end{array}$$

For example, consider the DNA string of length 10 as in

(2) $$\begin{array}{c} D\left[\;n\;\right]=\cdots \text{TTCACTAGCA}\cdots . \end{array}$$

The DNA string *D*[*n*] is converted into binary sequence *D*_*b*_[*n*] using EIIP representation as in

(3) $$\begin{array}{c} D_{b}\left[\;n\;\right]=\cdots 0.1335,0.1335,0.1340,0.1260\cdots . \end{array}$$

**Figure 1:**
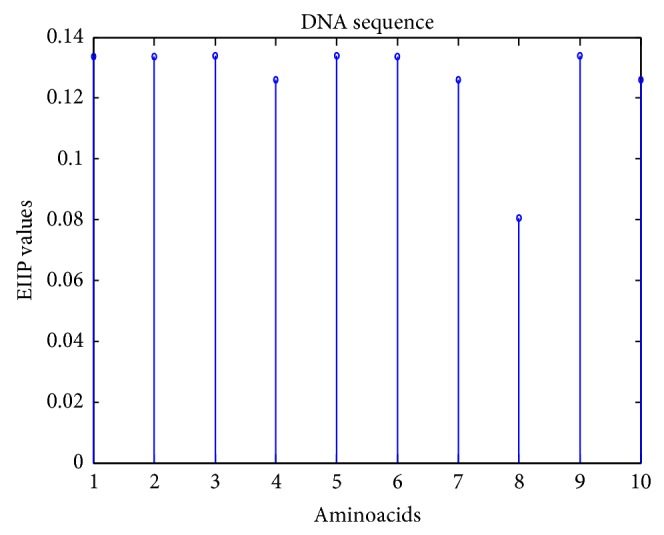
EIIP representation of a sample DNA sequence.

The sequence *D*_*b*_[*n*] obtained for (3) is shown in Figure 1.

**Figure 2:**
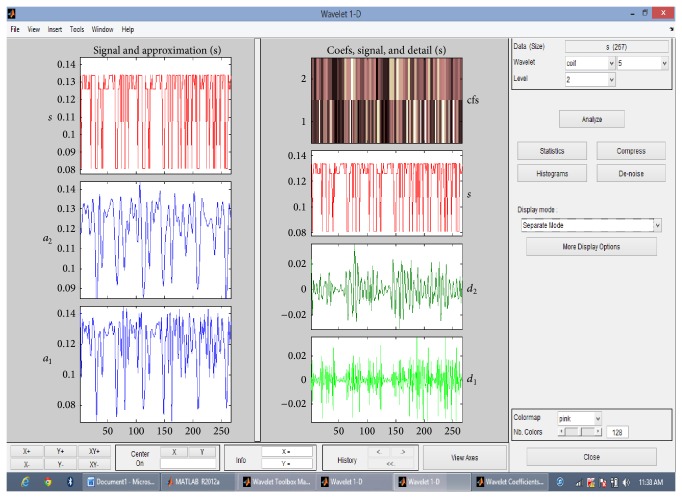
Approximate and detail coefficients for Coiflet 5 (level 2) using wavelet tool box.

**Figure 3:**
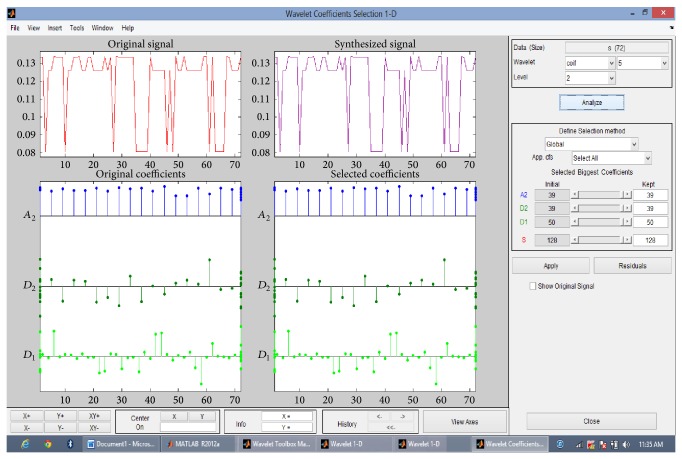
Coiflet 5 (level 2), wavelet coefficients selection using wavelet tool box.

**Figure 4:**
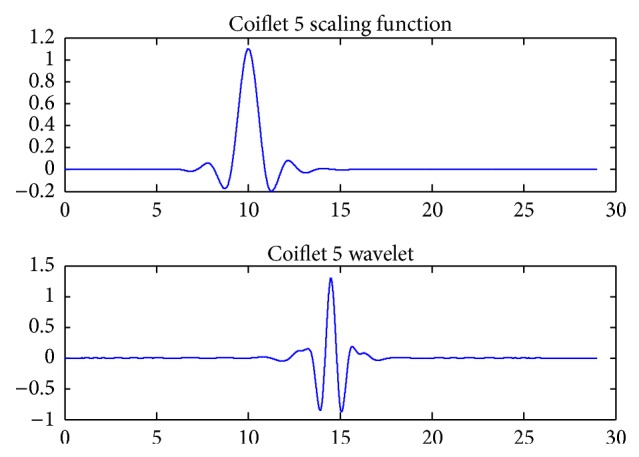
Scaling function and wavelet function for Coiflet 5.

Then the discrete wavelet transform of the DNA sequence under test is computed. The shape or choice of the mother wavelet depends on the properties of the signal we wish to analyze. Comparing the magnitude variations of the input DNA sequences with the characteristics of wavelets, it is concluded that Coiflet 5 (level 2) is suitable for analysis of input DNA sequences. The choice of the wavelet is justified with the use of wavelet tool box for a segment of DNA sequence as shown in Figures 2 and 3. The wavelet used is shown in Figure 4.

**Figure 5:**
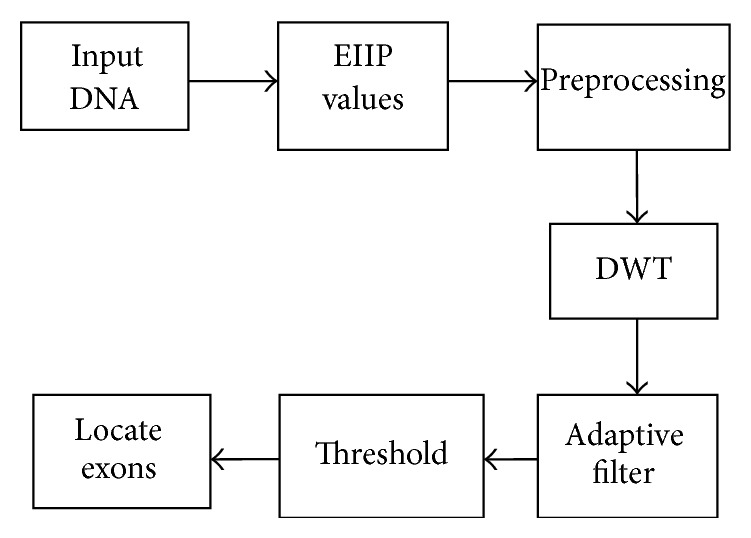
Block diagram for location of protein coding region using DWT.

It is known that the protein coding regions in a DNA sequence exhibit period-3 property producing larger amplitude coefficients for protein coding regions in transformed domain. Hence, the transform provides distinct energy concentrated areas in the time-frequency plane. The whole process of discrete wavelet transform based protein coding region identification scheme is shown in Figure 5.

The step-by-step process of our scheme for identification of exons is described as follows:

*Step 1.* The input sequence data are collected from the NCBI website.

*Step 2.* The DNA character sequences are converted into numeric sequences using EIIP representation.

*Step 3.* The numeric sequences are preprocessed using band pass filter to enhance the period-3 property of exons.

*Step 4.* Discrete wavelet transform is applied on the preprocessed signal to locate the protein coding regions.

*Step 5.* Adaptive filtering is done to remove the unwanted noise.

*Step 6.* The peaks of the filtered signal indicate the locations of the protein coding regions.

*Step 7.* Suitable thresholding is applied to locate the position of the protein coding regions.

## 4. Experimental Setup and Simulation Results

### 4.1. Experimental Studies

**Figure 6:**
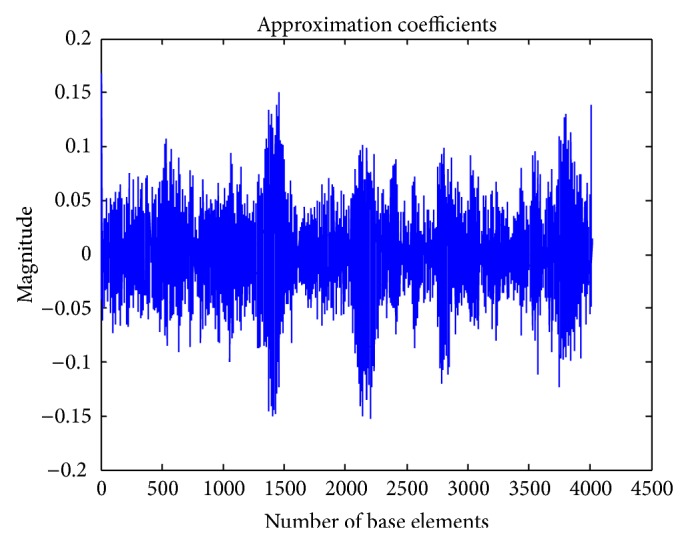
Approximate coefficients of DWT.

**Figure 7:**
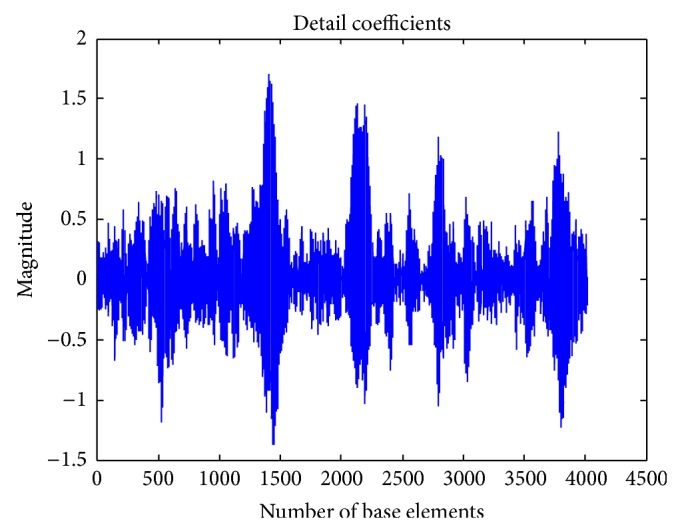
Detail coefficients of DWT.

**Figure 8:**
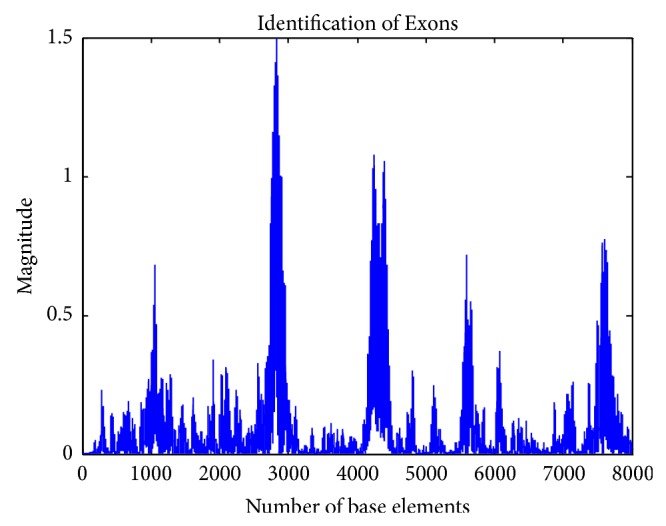
Output of the proposed system.

The proposed scheme based on discrete wavelet transformation is implemented in MATLAB Version 2013. The system on which the method was simulated was having 8 GB RAM with 64-bit operating system having i7 Processor. Eukaryotic DNA sequences are analysed to explain the performance of proposed system. For evaluation, gene* F56F11.4* of* Caenorhabditis elegans* chromosome III [Gene bank: AF099922] has been used since this has been used as a benchmark in literature. The approximate coefficients and detail coefficients of the wavelet transform are shown in Figures 6 and 7, respectively. The output obtained after filtering is shown in Figure 8. The peaks present in the output represent the protein coding regions.

### 4.2. Performance Evaluation

In this section, a detailed analysis of the protein coding region identification scheme has been done. The evaluation is carried out using the following metrics:

(4) SensitivityTPTP+FN,Specificity=TNTN+FP,Accuracy=TN+TPTN+TP+FN+FP,Positive Precision=TPTP+FP,Negative Precision=TNTN+FN,Error Rate=FP+FNTP+TN+FP+FN,False Discovery Rate=FPTP+FP,False Omission Rate=FNTN+FN,

where TP stands for True Positive, TN stands for True Negative, FN stands for False Negative, and FP stands for False Positive. These terms are defined in Table 2 and the values obtained in our experiment are summarized in Table 3.  Table 4 shows the comparison of evaluation metrics, sensitivity, specificity, and accuracy for various thresholds.  Table 5 shows the comparison of evaluation metrics for Error Rate, False Discovery Rate, and False Omission Rate for various thresholds.

**Figure 9:**
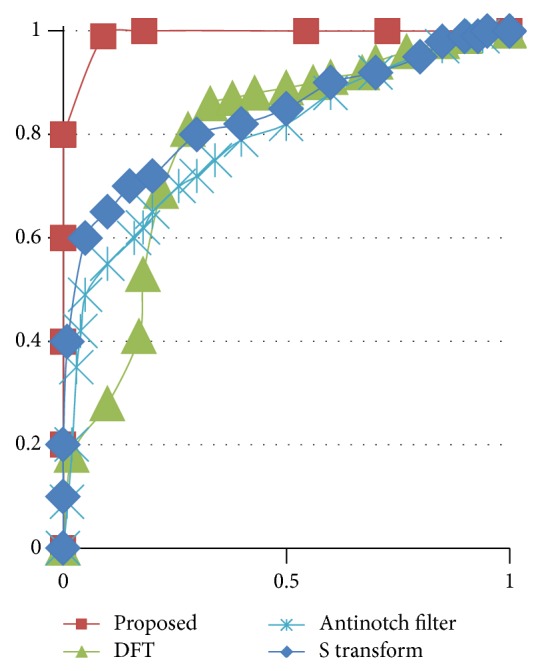
True Positive rate versus False Positive rate of the gene* F56F11.4*.

Calculating the area under the ROC curve (AUC) can be a better option for evaluating the performance of the method. ROC curves define the variations of True Positive rate with respect to False Positive rate under varying thresholds and the curve obtained for the proposed method is shown in Figure 9. If the AUC calculated is closer to 1.0, it is the effective method and if the AUC obtained is closer to 0.5 it is the least effective method. It is found that the AUC for the proposed method is 0.98. From this, the superiority of the method can be assessed.

It is customary to repeat the experiment for a number of samples for statistical analysis and validation of the scheme. It is learnt that the HMR 195 data set is the standard data set used for inputs in the problem of protein coding region identification and the same is taken as input in our experiment. This data set consists of 195 sequences comprising of 103 genes of human beings, 82 genes from Mouse, and 10 genes of Rat. The experiment has been conducted for 100 samples with both single and multiple exons from the data set. Evaluation metrics in literature were calculated in order to make a comparative study. The results are tabulated in Table 6 and are compared with the methods found in literature. The proposed method shows improved performance and thereby the superiority of the method is established.

### 4.3. Discussion

It is observed that, in the gene* F56F11.4*, it is difficult to detect the first coding region along the positions 929–1039 properly in the DFT spectral content method and antinotch filter. But the wavelet filtering approach detects that region as better than these two methods. Moreover, for 100 genes from HMR 195 data set, our method produced optimum performance. It is advantageous that the proposed method is not affected by window length constraints. This method provides time-frequency representation of output spectrum. Moreover, it produces a multiresolution view of the signal. This helps to analyse the output signal easily. This is a model independent method. Hence, there is no need for any training sample to predict. The proposed method is immune to background noise producing a maximum signal to noise ratio of 24.0839 db for the gene* AF092047*. This enables better discrimination between the coding and the noncoding regions. This also increases the accuracy.

## 5. Conclusion

In this paper, we discuss methods based on wavelet transformation of DNA sequences to identify protein coding regions in DNA sequences. The main aim of this paper is to increase the accuracy and also to reduce the noise as much as possible. The results showed that our method has ability to detect even the short coding region and it outperforms existing methods. We have applied the Coiflet 5 (level 2) to our problem. We have used the gene* F56F11.4* obtained from National Centre for Biotechnology Information and 100 genes from HMR 195 dataset as test samples. On analyzing the evaluation metrics obtained, it is found that the wavelet transformation methods proved to be better compared to the methods in literature in terms of sensitivity, specificity, and accuracy. Positive and Negative Precision values are evaluated for wavelet method and it is found to be good. Negative evaluation metrics such as Error Rate, False Discovery Rate, and False Omission Rate are evaluated and showed good performance in the proposed method. Hence, evaluation parameters are found to be the best and this approach provides an effective and efficient approach to identify protein coding regions in DNA sequences. In addition, it is independent of the window length constraint and robust to background noise present in the DNA sequence.

## Figures and Tables

**Table 1:** EIIP values of nucleotides.

Nucleotide	EIIP
A	0.1260
G	0.0806
T	0.1335
C	0.1340

**Table 2:** Definition of the terms.

Experimental outcome	Condition as determined by the Standard of Truth	
	Positive	Negative
Positive	TP	FP
Negative	FN	TN

**Table 3:** Evaluation metrics of the gene *F56F11.4*.

Threshold	TP	FP
0.25	100%	40%
0.3	100%	20%
0.35	100%	0
0.4	100%	0
0.45	100%	0
0.5	100%	0
0.55	80%	0
0.6	80%	0
0.65	80%	0

**Table 4:** Evaluation metrics of the gene *F56F11.4*.

Threshold	Sn (%)	Sp (%)	Ac (%)	PP (%)	Np (%)
0.25	100	60	80	71.42	100
0.3	100	80	90	83.33	100
0.35	100	100	100	100	100
0.4	100	100	100	100	100
0.45	100	100	100	100	100
0.5	100	100	100	100	100
0.55	80	100	90	100	80
0.6	80	100	90	100	80
0.65	80	100	90	100	80

**Table 5:** Evaluation metrics of the gene *F56F11.4*.

Threshold	ER	FDR	FOR
0.25	20%	28.57%	0%
0.3	10%	16.66%	0%
0.35	0%	0%	0%
0.4	0%	0%	0%
0.45	0%	0%	0%
0.5	0%	0%	0%
0.55	10%	0%	16.66%
0.6	10%	0%	16.66%
0.65	10%	0%	16.66%

**Table 6:** Evaluation metrics of 100 genes from HMR 195 dataset.

Method	Average sensitivity	Average specificity	Average accuracy
Multiresolution	**0.89**	**0.88**	**0.86**
S transform	0.88	0.88	0.85
SONF	0.89	0.86	0.84
DFT	0.86	0.83	0.84
Antinotch filter	0.81	0.82	0.82
HMM	0.82	0.84	0.83
